# Comprehensive analysis of ferroptosis-related gene signatures as a potential therapeutic target for acute myeloid leukemia: A bioinformatics analysis and experimental verification

**DOI:** 10.3389/fonc.2022.930654

**Published:** 2022-08-11

**Authors:** Zhiyuan Zheng, Xiaoying Hong, Xiaoxue Huang, Xiandong Jiang, He Jiang, Yingying Huang, Wei Wu, Yan Xue, Donghong Lin

**Affiliations:** ^1^ Medical Technology and Engineering College of Fujian Medical University, Fuzhou, China; ^2^ Medical Technology Experimental Teaching Center of Fujian Medical University, Fuzhou, China; ^3^ Department of Radiation Oncology, Fujian Medical University Union Hospital, Fuzhou, China

**Keywords:** ferroptosis, acute myeloid leukemia, gene signatures, prognosis, The Cancer Genome Atlas, sulfasalazine

## Abstract

**Background:**

Ferroptosis plays an important role in the development of acute myeloid leukemia (AML); however, the exact role of ferroptosis-related genes in the prognosis of AML patients is unclear.

**Methods:**

RNA sequencing data and the clinicopathological characteristics of AML patients were obtained from The Cancer Genome Atlas database, and ferroptosis-related genes were obtained from the FerrDb database. Cox regression analysis and least absolute shrinkage and selection operator analysis were performed to identify ferroptosis-related gene signatures. Gene Ontology (GO), Kyoto Encyclopedia of Genes and Genomes (KEGG), and single-sample gene set enrichment analysis (ssGSEA) were performed to explore the biological functions of the ferroptosis-related genes. Finally, ferroptosis of AML cells was induced by erastin and sulfasalazine to detect the changes in the expression of relevant prognostic genes and explore the underlying mechanisms using quantitative real-time polymerase chain reaction (qRT-PCR).

**Results:**

Seven ferroptosis-related gene signatures (*SOCS1*, *ACSF2*, *MYB*, *EIF2AK4*, *AIFM2*, *SLC7A11*, and *GPX4*) were identified in the training group. Kaplan-Meier and Cox regression analyses confirmed that risk score was an independent prognostic predictor of AML in the training and validation groups (*P*<0.05). Further, functional enrichment analysis revealed that seven ferroptosis-related genes were associated with many immune-related biological processes. Most importantly, erastin and sulfasalazine can induce the ferroptosis of AML cells. Overall, *SLC7A11* and the SLC7A11/xCT-GSH-GPX4 pathway may be the respective key gene and potential regulatory pathway in erastin- and sulfasalazine-induced ferroptosis of AML cells.

**Conclusions:**

A novel signature involving seven ferroptosis-related genes that could accurately predict AML prognosis was identified. Further, the Food and Drug Administration-approved drug, sulfasalazine, was demonstrated for the first time to induce the ferroptosis of AML cells. *SLC7A11* and the SLC7A11/xCT-GSH-GPX4 pathway may be the respective key gene and underlying mechanism in this process, ultimately providing new insights into the strategies for the development of new AML therapies.

## Introduction

Acute myeloid leukemia (AML) is a hematological malignancy caused by clonal malignant proliferation of myeloid blasts in the hematopoietic system (1). AML is the most common type of leukemia in adults and a leading cause of cancer-related deaths (2). Despite recent advances in the treatment modalities for leukemia, the overall prognosis of AML remains unsatisfactory owing to its complex molecular mechanisms and tumor microenvironment (3). Therefore, new and effective therapeutic strategies are urgently required to improve the prognosis of AML patients.

Ferroptosis is a recently discovered type of programmed cell death characterized by iron-dependent accumulation of lipid reactive oxygen species (ROS). Ferroptosis was first proposed by Dixon etal. (4) as a new method of cell death regulation. Based on previous studies, ferroptosis is involved in cardiovascular disease, ischemia-reperfusion injury, and cancer development (5). Leukemia cells are highly sensitive to ferroptosis inducers (6); therefore, ferroptosis may be closely related to the development of leukemia. Researchers have constructed corresponding prognostic prediction models based on the expression levels of ferroptosis-related genes in AML patients, which are available in public databases (7, 8), to identify potential therapeutic targets and mechanisms. Then, the corresponding predictive model needs further experimental studies to confirm whether some key genes in the model are involved in the regulation of ferroptosis in AML.

By downloading the RNA-seq expression profiles of AML patients and their corresponding clinical data from The Cancer Genome Atlas (TCGA), we constructed a ferroptosis-related gene signature associated with AML prognosis. Due to the close relationship between ferroptosis and the immune microenvironment (9), we initially explored the link between this model and the immune microenvironment. Further, we confirmed that the ferroptosis inducer, erastin, can induce the ferroptosis of AML cells and for the first time, revealed that sulfasalazine, a drug clinically used to treat rheumatoid arthritis (10), can induce the ferroptosis of AML cells. Finally, we performed quantitative real-time polymerase chain reaction (qRT-PCR) to detect changes in the expression of molecules in ferroptosis-related gene signatures and sought to identify new potential therapeutic targets and mechanisms.

## Materials and methods

### Data collection

In this study, the expression data and clinical data of AML patients were collected from TCGA. Genes were encoded according to the GRCh38 version. The following clinical information was collected: age; sex; cytoplasmic nucleophosmin [NPMc] mutations; isocitrate dehydrogenase 1 [IDH1] R132, IDH1 R140, and FMS-like tyrosine kinase 3 [FLT3], and French-American-British [FAB] subtype; cytogenetic risk category; and follow-up time. The initially obtained 151 samples were screened according to the following criteria: 1) samples with complete survival data and survival for more than 30 days; and 2) samples derived from the bone marrow of patients diagnosed with AML based on relevant diagnostic criteria. After screening, 130 AML patients were included in the study and were randomized divided into a training group (=94) and a validation group (=36) using the “caret” package in R (**Supplementary File 1**). The 245 ferroptosis-related gene sets (**Supplementary File 2**) were obtained from the FerrDb database, which is the world’s first database dedicated to ferroptosis regulators and markers (11).

### Establishment of prognostic ferroptosis-related gene signatures

The genes associated with the overall survival of AML patients were obtained *via* univariate analysis. Thereafter, the “glmnet” package in the R software was used to perform least absolute shrinkage and selection operator (LASSO) regression to avoid overfitting. Finally, a multivariate Cox regression was performed to establish a prognostic gene signature with the lowest Akaike information criterion (AIC) value (12). The risk score for each AML patient in the model was calculated based on its corresponding coefficient and the gene expression level using the following formula:


βmRNA1×ExpressionmRNA1+βmRNA2×ExpressionmRNA2+...+βmRNAn×ExpressionmRNAn.

### Evaluation of the prediction ability of the ferroptosis-related gene signatures

Patients in the training and validation sets were divided into low- and high-risk sets based on the median risk score of the training set. Survival curves were generated using the Kaplan-Meier method, and the predictive performance of the model was evaluated using receiver operating characteristic (ROC) curve. Subsequently, t-distributed stochastic neighbor embedding (t-SNE) (13) and principal component analysis (PCA) (14) were performed using the Rtsne package in the R software to reduce and visualize the dimensions of different ferroptosis statuses based on the low- and high-risk groups.

### Functional enrichment analysis

The limma package in R was employed to identify differentially expressed genes (DEGs) between the high- and low-risk groups using log fold change (FC) > 0.5 and a false discovery rate (FDR)< 0.05 as indicators of statistical significance. Subsequently, Gene Ontology (GO) and Kyoto Encyclopedia of Genes and Genomes (KEGG) analyses of DEGs were performed using the “cluster profiler” R package, and the results were visualized using the “gg plot2” R package. To further explore the relationship between the ferroptosis-related gene signatures and immune microenvironment, single-sample gene set enrichment analysis (ssGSEA) was used to calculate the infiltration scores of 13 immune-related pathways and 16 immune cells (15) using the “gsva package” (16) in R software (**Supplementary File 3**).

### Cell culture

The human AML lines, Kasumi-1 (#MC-1087) and HL-60 (#CL-0110), were purchased from the Procell Life Technology Company (Wuhan, China). Cells were cultured in RPMI-1640 medium containing 10% fetal bovine serum (FBS) (Capricorn, #FBS-12A) in a humidified incubator at 37°C with 5% CO_2_. The old cell culture medium was replaced with fresh medium every two days.

### Reagents

Erastin (#GC16630), sulfasalazine (#GC16868), necrostatin-1 (#GC11008), Ferrostatin-1 (Fer-1) (#GC10380), chloroquine (#GC10295), deferoxamine mesylate (DFO) (#GC13554), and Z-VAD-FMK (#GC12861) were purchased from GlpBio.

### Cell viability assay

Cell viability was determined using the MTS cell proliferation colorimetric assay kit (Promega, #G3580). Kasumi-1 and HL-60 cells were seeded in 96-well platess at a density of 10000 cells per well and cultured in a humidified incubator at 37°C with 5% CO2 for 24 h. Cells were then treated with specific concentrations of reagents and incubated for 24 h in a humidified incubator at 37°C with 5% CO_2_. Subsequently, 20 µL of MTS solution were added to each well of the plate and incubated for 4 h at 37°C with 5% CO_2_. Finally, the absorbance (OD value) at wavelengths of 490/630 nm was measured using a microplate reader (Thermo, #1410101). All experiments were performed in triplicate and repeated three times.

### Lipid peroxidation assay

The MDA level in the cells was detected using a Micro Malondialdehyde Assay Kit (#BC0025). The cells were homogenized with lysis buffer, and MDA in the sample was reacted with thiobarbituric acid (TBA) to form the MDA-TBA adduct. The associated change in the OD value was subsequently measured by colorimetry. For the specific operation process, please refer to **Supplementary File 4**.

BODIPY™ 581/591 C11 (#D3861; Invitrogen) was used to detect ROS in cells. Treated cells were incubated with 1 μM BODIPY 581/591C11 for 30 min, washed with phosphate-buffered saline (PBS), and analyzed using an Accuri C6 Flow Cytometer (BD, USA).

### Redox metabolism assay

The redox metabolites were detected using a glutathione peroxidase 4 assay kit (Bioss, #AK091) and a Micro Reduced Glutathione (GSH) Assay Kit (Solarbio, #BC1175). All reagents were used in accordance with the manufacturer’s instructions. The cells were homogenized using lysis buffer, and the redox analyte in the test sample reacted with the reagents to form adducts that could be quantified *via* colorimetry.

### Quantitative real-time polymerase chain reaction

Total RNA from Kasumi-1 and HL-60 cells treated with erastin or sulfasalazine was extracted using TRIzol reagent (Vazyme, #R701-01-AA). The concentration and purity of all RNA samples were determined using the absorbance ratios at 260/280 nm and 260/230 nm, respectively. cDNA was synthesized from 1 µg of total RNA using Hifair TMII First-Strand cDNA Synthesis Super Mix (Yeasen, #11123ES60). Subsequently, real-time PCR analysis was performed on a GENTIER-96 real-time fluorescent quantitative PCR system (Tianlong, China) using the SYBR Green master mix (Yeasen, #11202ES08). The threshold cycle (Ct) values for each gene were normalized to that of GAPDH. The primers used for real-time PCR are listed in **Supplementary File 5** and were purchased from Shangya Biotechnology (Fuzhou, China).

### Western blot analysis

The proteins from Kasumi-1 and HL-60 cells treated with sulfasalazine were lysed in Radio Immunoprecipitation Assay (RIPA) buffer containing phosphatase and protease inhibitor. After centrifugation at 4 °C, the proteins in the supernatant were collected and the protein concentration was determined by bicinchoninic acid (BCA) protein kit (Sigma, #71285-M). Forty micrograms of protein were separated on 10% sodium dodecyl sulfate-polyacrylamide gel electrophoresis (SDS-PAGE) and transferred to polyvinylidene difluoride (PVDF) membranes. Subsequently, the PVDF membranes were blocked with 5% non-fat milk in TBST (TBS buffer containing 0.1% Tween 20) to prevent nonspecific binding and incubated with primary antibodies GAPDH (ImmunoWay, #YN5585) and SLC7A11 (Abcam, #ab175186) overnight at 4 °C. After washing with TBST, the membranes were incubated with anti-horseradish peroxidase-linked IgG secondary antibody (ImmunoWay, #RS0002). Blots were developed with an ECL kit (Meilunbio, #MA0186).

### Statistical analysis

The data are presented as mean ± standard deviation (SD). Differences between groups were analyzed using the chi-square tests, Mann-Whitney U test, Student’s *t*-test, or one-way analysis of variance (ANOVA). All statistical analyses were performed with R version 4.0.1 or GraphPad Prism 8, and all experiments were repeated at least three times. Statistical significance was set at *P*<0.05.

## Results

### Acquisition of ferroptosis-related mRNA

**Figure 1:**
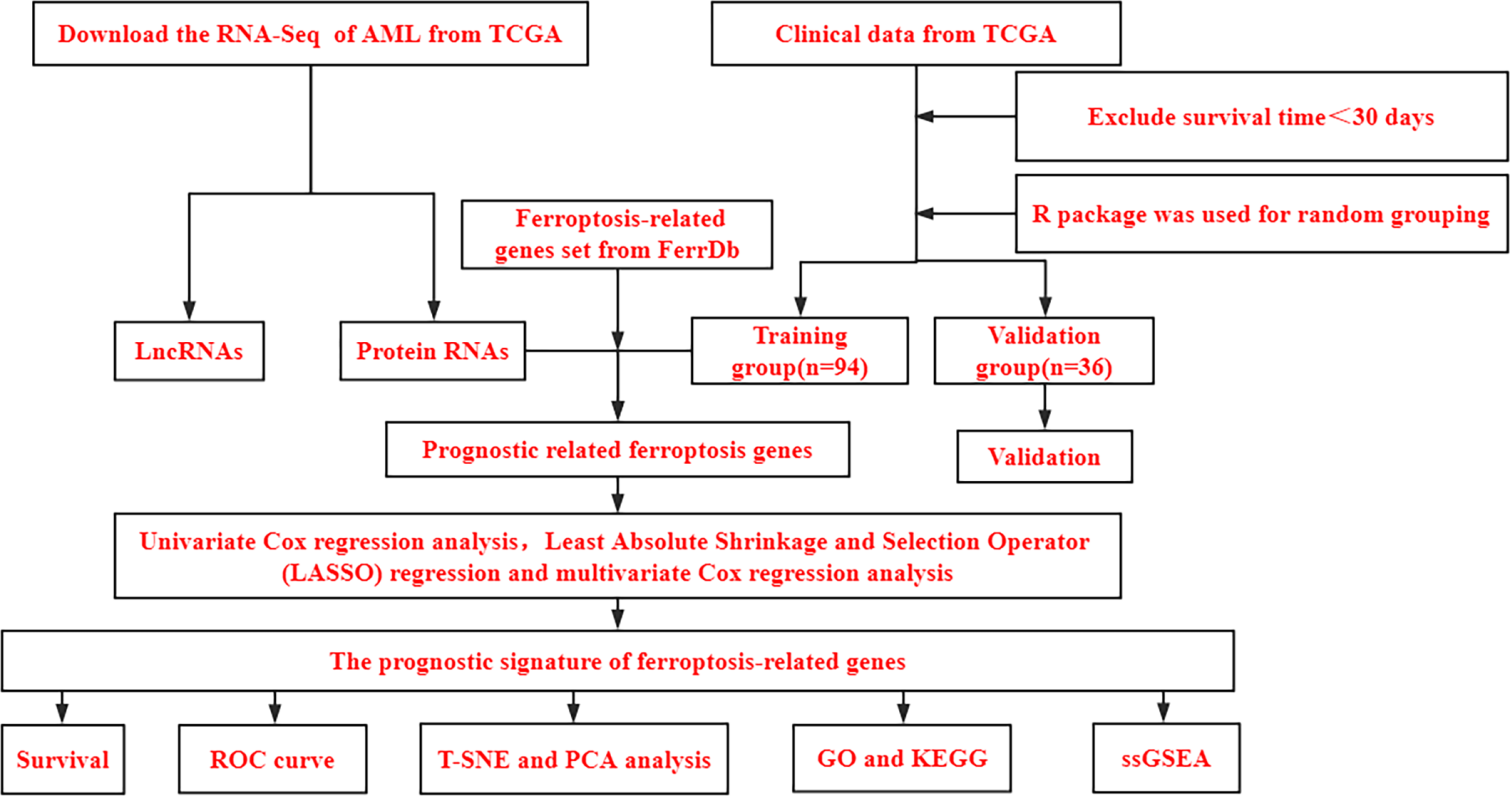
Bioinformatics Analysis Flowchart. TCGA, The Cancer Genome Atlas database; AML, Acute myeloid leukemia; PCA, principal component analysis; t-SNE, *t*-distributed stochastic neighbor embedding; GO, Gene Ontology; KEGG, Kyoto Encyclopedia of Genes and Genomes; ssGSEA, single-sample gene set enrichment analysis.

**Figure 1** shows a flowchart of the bioinformatics analysis performed in this study. We retrieved 245 ferroptosis-related genes from FerrDb (**Supplementary File 2**) and intersected them with the gene expression profiles of TCGA-LAML through the “limma package” in R language, and finally obtained the expression of 238 ferroptosis-related genes. The clinicopathological characteristics of the 130 AML patients are presented in **Table 1**. A comparison between the training and validation groups revealed no differences in the clinicopathological characteristics.

**Table 1:** Characteristics of patients with AML.

Characteristics	Patients (N,%)	(N,%)		*P*
		Training group (n=94)	Validation group (n=36)	
Sex Female Male	61 (46.9) 69 (53.1)	43 (45.7) 51 (54.3)	18 (50.0) 18 (50.0)	0.664
Age,years ≤55 >55	65 (50.0) 65 (50.0)	48 (51.1) 46 (48.9)	17 (47.2) 19 (52.8)	0.695
FAB subtype M0 M1 M2 M3 M4 M5 M6 M7	12 (9.2) 31 (23.8) 32 (24.6) 13 (10.0) 27 (20.8) 12 (9.2) 2 (1.6) 1 (0.8)	8 (8.5) 19 (20.2) 22 (23.4) 9 (9.6) 23 (24.5) 10 (10.6) 2 (2.1) 1 (1.1)	4 (11.1) 12 (33.3) 10 (27.8) 4 (11.1) 4 (11.1) 2 (5.6) 0 (0) 0 (0)	0.514
Cytogenetics risk category Favorable Intermediate/Normal Poor Unknow	29 (22.3) 72 (55.4) 27 (20.8) 2 (1.5)	21 (22.4) 52 (55.3) 19 (20.2) 2 (2.1)	8 (22.2) 20 (55.6) 8 (22.2) 0 (0)	0.931
FLT3 mutation Positive Negative Unknow	33 (25.4) 93 (71.5) 4 (3.1)	23 (24.5) 69 (73.4) 2 (2.1)	10 (27.8) 24 (66.7) 2 (5.5)	0.468
IDH1 R132 Positive Negative Unknow	13 (10) 116 (89.2) 1 (0.8)	10 (10.6) 84 (89.4) 0 (0)	3 (8.3) 32 (88.9) 1 (2.8)	0.413
IDH1 R140 Positive Negative Unknow	11 (8.5) 117 (90.0) 2 (1.5)	9 (9.6) 84 (89.3) 1 (1.1)	2 (5.6) 33 (91.7) 1 (2.8)	0.519
NPMc Positive Negative Unknow	31 (23.8) 98 (75.4) 1 (0.8)	26 (27.7) 67 (71.3) 1 (1.0)	5 (13.9) 31 (86.1) 0 (0)	0.179
Follow-up state Alive Died	51 (39.2) 79 (60.8)	36 (38.3) 58 (61.7)	15 (41.7) 21 (58.3)	0.841

FLT3, FMS-like tyrosine kinase 3; NPMc, nucleophosmin mutations; FAB, French-American-British; IDH1, isocitrate dehydrogenase 1.

### Development and validation of the prognostic ferroptosis-related gene signature

**Figure 2:**
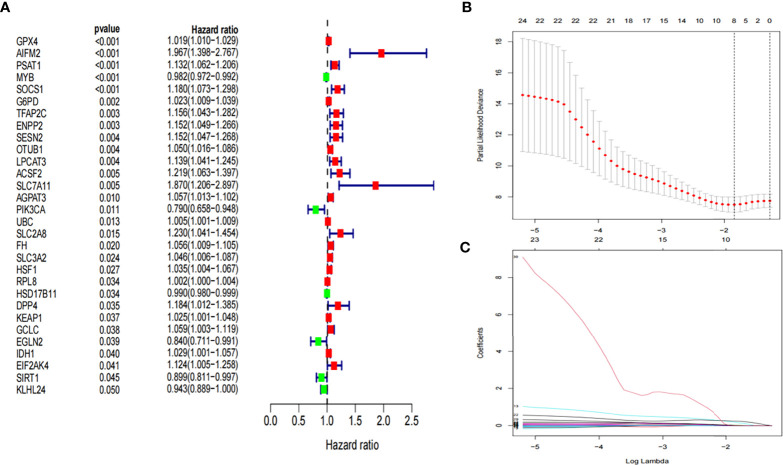
Development of prognostic ferroptosis-related genes. **(A)** Forest plot of univariate Cox regression identified 30 ferroptosis-related genes associated with overall survival. **(B, C)** LASSO Cox regression was performed to identify ferroptosis-related genes closely associated with prognosis of AML. LASSO, Least Absolute Shrinkage and Selection Operator; AML, Acute myeloid leukemia;.

A ferroptosis-related gene signature was constructed based on the training group. First, 30 ferroptosis-related genes that were significantly associated with overall survival (OS) were obtained using univariate Cox analysis (*P*< 0.05, **Figure 2A**). Thereafter, based on LASSO regression, eight ferroptosis-related genes with a repetition rate > 900 times in 1000 replacement samples were screened out (**Figures 2B, C**). Finally, a multivariate Cox regression analysis was performed and a gene signature was constructed based on the lowest AIC value, which led to seven ferroptosis-related gene signatures for the prediction of prognosis (**Table 2**). The median risk score, which was used to divide patients into low-risk (n = 47) or high-risk (n = 47) groups, was calculated as follows:


$$\begin{array}{l} Risk\;score=0.2283\times Expression\;ACSF2+0.1089\times Expression\;SOCS1-0.0140\times \\ Expression\;MYB+0.8615\times Expression\;SLC7A11+0.0217\times Expression\;GPX4+0.4538\times \\ Expression\;AIFM2+0.1988\times Expression\;EIF2AK4. \end{array}$$

**Table 2:** Seven ferroptosis-related messenger RNAs(mRNAs) detected using multivariable Cox regression analysis.

ID	Coef	HR	HR.95L	HR.95H	*P* value
ACSF2	0.2283	1.2565	1.0674	1.4790	0.00607285
SOCS1	0.1089	1.1150	0.9875	1.2589	0.07876917
MYB	-0.0140	0.9860	0.9740	0.99813	0.02390114
SLC7A11	0.8615	2.3666	1.4810	3.7818	0.00031567
GPX4	0.0217	1.0220	1.0080	1.0361	0.00185572
AIFM2	0.4538	1.5742	1.0330	2.3991	0.03476102
EIF2AK4	0.1988	1.2199	1.0649	1.3975	0.00414455

coef, coefficient; HR, hazard ratio.

**Figure 3:**
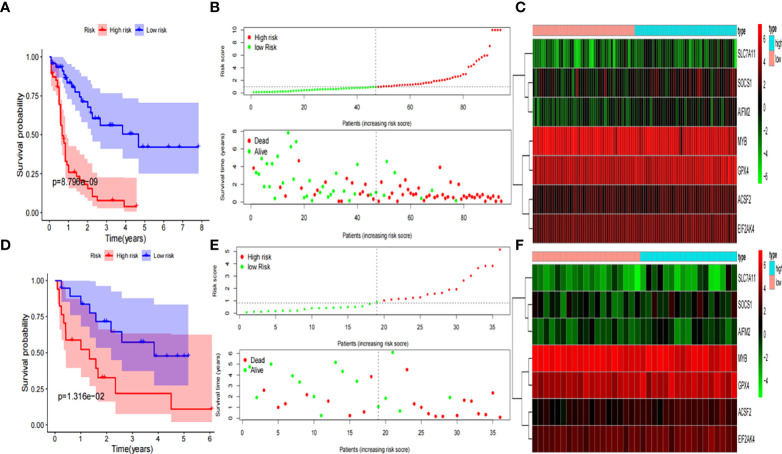
Development and validation of prognostic ferroptosis-related genes signature. **(A)** Kaplan-Meier curve, **(B)** risk score and survival status, and **(C)** heatmap for training group. **(D)** Kaplan-Meier curve, **(E)** risk score and survival status, and **(F)** heatmap for validation group.

The Kaplan-Meier curve showed that the probability of death significantly increased for patients with high-risk scores in the training group (*P*< 0.001, **Figure 3A**). As the risk score increased, the risk of death increased and the duration of survival decreased (**Figure 3B**). The risk heatmap clearly revealed the expression of different mRNAs in the low- and high-risk groups (**Figure 3C**). Thereafter, our results were confirmed using the validation group. OS was found to significantly differ between the low- and high-risk groups (*P<* 0.05, **Figures 3D, E**). The heatmap also revealed the expression of the corresponding genes (**Figure 3F**), which validated the reliability of our results. Scatter plots and the Mann-Whitney U test were employed to better reflect the expression differences of seven ferroptosis-related genes between the low- and high- risk groups in the entire cohort (**Supplementary Figure 1**). The mRNA expression levels of *ACSF2*, *SOCS1*, *MYB*, *EIF2AK4*, *AIFM2*, *SLC7A11*, and *GPX4* were found to significantly differ in the entire cohort (All *P<* 0.05). By exploring the distribution of the low- and high-risk groups using t-SNE (**Supplementary Figures 2A, B**) and PCA (**Supplementary Figures 2C, D**), we intuitively perceived that AML patients could be better differentiated based on prognosis, as indicated by the mRNAs related to ferroptosis.

### Independent prognostic analysis of OS

**Figure 4:**
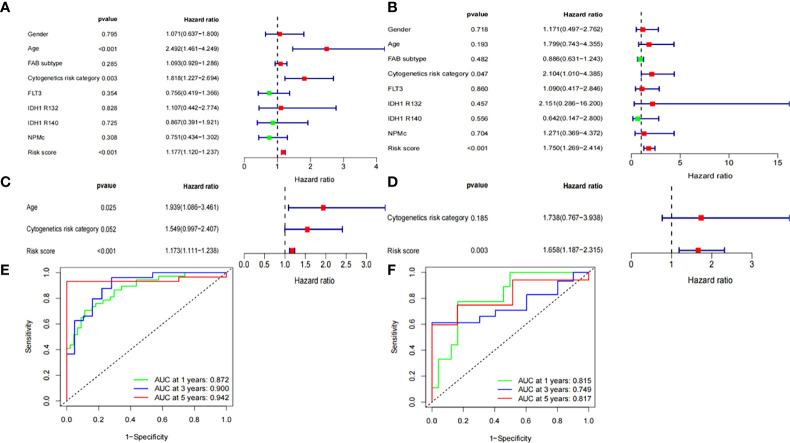
Independent prognostic factors for AML overall survival. Univariate Cox regression analysis in **(A)** training group and **(B)** validation group, respectively. Multivariate Cox regression analysis in **(C)** training group and **(D)** validation group, respectively. Receiver operating characteristic curve (ROC) analysis of risk scores based on 1-, 3-, and 5-year OS in **(E)** training group and **(F)** validation group, respectively.

We assessed whether the clinical characteristics and risk scores were independent prognostic factors for OS using univariate and multivariate Cox regression analyses. Risk score was identified as an independent prognostic predictor of OS in both the validation and training groups based on univariate and multivariate Cox regression analysis (**Figures 4A–D**). The time-dependent ROC curve analysis also revealed that the AUC for the risk scores for 1-, 3-, and 5-year OS were 0.872, 0.900, and 0.945 in the training group (**Figure 4E**), and 0.815, 0.749, and 0.817 in the validation group (**Figure 4F**), respectively. This result further suggests that ferroptosis-related gene signatures have higher diagnostic performance in predicting the prognosis of patients with AML.

### Functional enrichment analysis of low- and high-risk patient groups based on GO, KEGG, and ssGSEA

**Figure 5:**
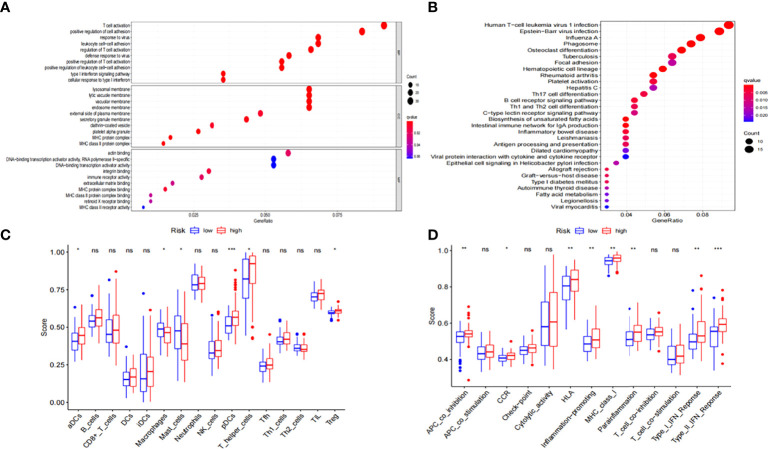
Functional enrichment analysis of the DEGs between low-risk and high-risk groups. **(A)** GO and **(B)** KEGG enrichment analyses of DEGs in the TCGA-LAML cohort. **(C)** ssGSEA scores of 16 immune cells and **(D)** 13 immune-related functions in the TCGA-LAML cohort. DEGs, differentially expressed genes; GO, Gene Ontology; KEGG, Kyoto Encyclopedia of Genes and Genomes; ssGSEA, Single-sample gene set enrichment analysis; NS: not significant; *P < 0.05; **P < 0.01; ***P < 0.001.

To further elucidate the potential biological functions of the seven ferroptosis-related genes, we performed GO and KEGG pathway analyses based on the DEGs between the low- and high-risk groups in the entire TCGA cohort. Human T-cell leukemia virus 1 infection and many immune-related pathways were identified to be enriched (**Figures 5A, B**).

We used ssGSEA to further estimate immune cell infiltration in patients with AML to explore the differences in immune status between the low- and high-risk groups. Macrophages and mast cells were found to considerably increase in the low-risk group (**Figure 5C**) while activated dendritic cells (aDCs), T helper cells, plasmacytoid DCs (pDCs), regulatory T cells (Tregs), chemokine receptors (CCR), APC co-inhibition, human leukocyte antigen (HLA), MHC-class-I, para-inflammation, inflammation-promoting, and type-I and type-II interferon responses were significantly increased in the high-risk group (**Figures 5C, D**).

### Erastin and sulfasalazine inhibit the cell viability of Kasumi-1 and HL-60

**Figure 6:**
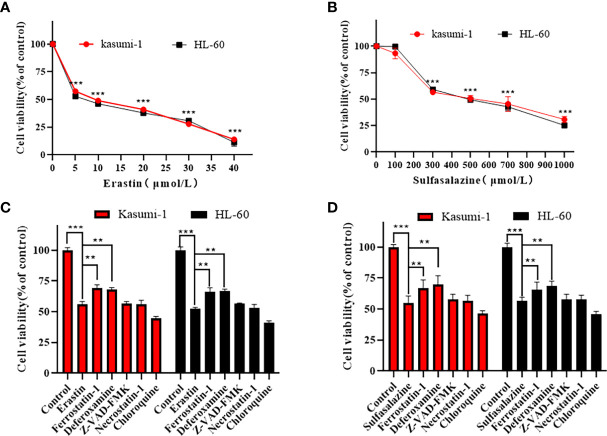
Erastin and sulfasalazine induce ferroptosis in Kasumi-1 and HL-60 cells. **(A)** The viability of Kasumi-1 and HL-60 cells treated with different concentrations of erastin for 24 h was determined using the MTS method. **(B)** The viability of Kasumi-1 and HL-60 cells treated with different concentrations of sulfasalazine for 24 h was determined using the MTS method. **(C)** Kasumi-1 and HL-60 cells treated with normal or 5µM erastin were cultured with Ferrostatin-1(2 µM), deferoxamine (100 µM), necrostatin-1 (10 µM), Z-VAD-FMK (10 µM) or chloroquine (25 µM) for 24 h, and cell viability was detected using the MTS assay. **(D)** Kasumi-1 and HL-60 cells treated with normal or 300µM sulfasalazine were cultured with Ferrostatin-1(2 µM), deferoxamine (100 µM), necrostatin-1 (10 µM), Z-VAD-FMK (10 µM) or chloroquine (25 µM) for 24 h, and cell viability was detected using the MTS assay. **P < 0.01; ***P < 0.001.

Kasumi-1 and HL-60 cells were treated with different concentrations of the ferroptosis inducer, erastin, and the xCT system inhibitor, sulfasalazine, for 24 h. Based on the MTS results in **Figure 6A, B**, both erastin and sulfasalazine inhibited the proliferation of Kasumi-1 and HL-60 cells in a dose-dependent manner. When the drug concentration reached 5 µM and 300 µM, respectively, the growth inhibitory effects of erastin and sulfasalazine on the two AML cell lines were statistically significant.

### Erastin and sulfasalazine induce the ferroptosis of Kasumi-1 and HL-60 cells

**Figure 7:**
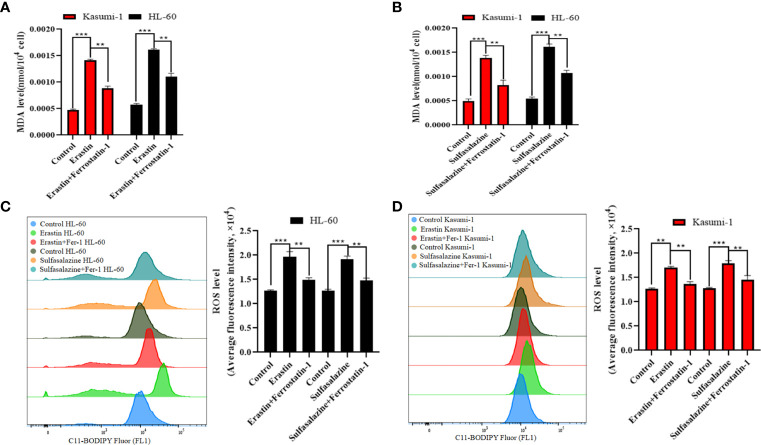
Detection of lipid peroxidation levels in Kasumi-1 and HL-60 cells after 24 h treatment with 5 µM erastin or 300 µM sulfasalazine. **(A)** MDA levels were tested in Kasumi-1 and HL-60 cells cultured for 24 h under normal, 5 µM erastin or 5 µM erastin+2 µM Ferrostatin-1 treatment conditions. **(B)** MDA levels were tested in Kasumi-1 and HL-60 cells cultured for 24 h under normal, 300 µM sulfasalazine or 300 µM sulfasalazine+2 µM Ferrostatin-1 treatment conditions. **(C)** Flow cytometry was used to detect ROS levels in HL-60 cell cultured for 24 h after normal, 5 µM erastin or 5 µM erastin+2 µM Ferrostatin-1 and normal, 300 µM sulfasalazine or 300 µM sulfasalazine+2 µM Ferrostatin-1 treatment conditions. **(D)** Flow cytometry was used to detect ROS levels in Kasumi-1 cell cultured for 24 h after normal, 5 µM erastin or 5 µM erastin+2 µM Ferrostatin-1 and normal, 300 µM sulfasalazine or 300 µM sulfasalazine+2 µM Ferrostatin-1 treatment conditions. **P < 0.01; ***P < 0.001.

To further elucidate the mechanism of AML cell death, subsequent experiments were performed using 5 µM erastin and 300 µM sulfasalazine. First, four types of cell death inhibitors, including ferroptosis inhibitors Fer-1 and DFO, apoptosis inhibitor Z-VAD-FMK, necroptosis inhibitor necrostatin-1, and autophagy inhibitor chloroquine, were applied to erastin- and sulfasalazine-treated AML cells. However, the inhibition of Kasumi-1 and HL-60 cell viability induced by erastin and sulfasalazine was only reversed by the ferroptosis inhibitors (Fer-1 and DFO) (**Figures 6C, D**). Ferroptosis is a novel regulated mode of cell death, and cellular abnormalities are caused by lipid peroxidation, particularly the accumulation of malondialdehyde (MDA) and lipid-based ROS, ultimately leading to oxidative stress. Therefore, by examining the ROS and MDA levels, we found that erastin and sulfasalazine induced increases in both MDA (**Figures 7A, B**) and ROS levels (**Figures 7C, D**) in Kasumi-1 and hl-60 cells, and could rescued by the ferroptosis inhibitor (Fer-1). These results indicate that erastin and sulfasalazine induced the ferroptosis of Kasumi-1 and HL60 cells.

### Expression of seven prognostic ferroptosis-related genes in Kasumi-1 and HL-60 after 24 h of treatment with 5 µM erastin and 300 µM sulfasalazine

**Figure 8:**
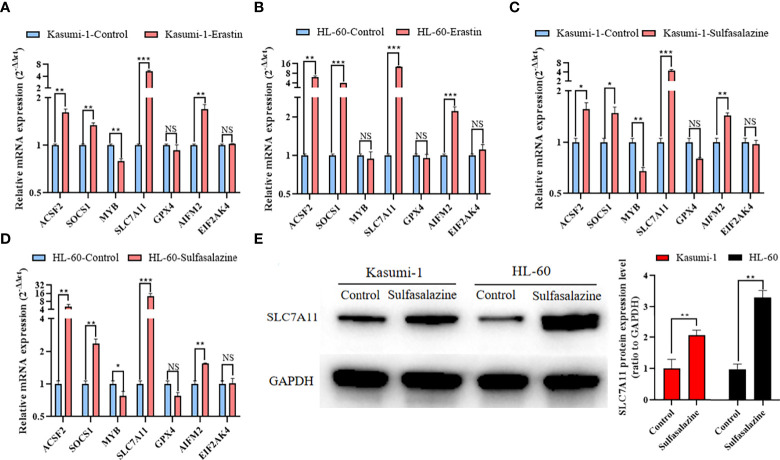
Expression of 7 prognostic ferroptosis-related genes in Kasumi-1 and HL-60 cells after 24 h treatment with 5 µM erastin and 300 µM sulfasalazine. qRT-PCR detection of mRNA expression levels of seven prognostic ferroptosis-related genes in **(A, C)** Kasumi-1 and **(B, D)** HL-60 cells treated with 5 mM erastin or 300 mM sulfasalazine. **(E)** SLC7A11 protein expression level in Kasumi-1 and HL-60 cells cultured for 24 hours under normal or 300 µM sulfasalazine treatment conditions. GAPDH mRNA and protein expressions were used as the loading control for qRT-PCR and Western blot. NS: not significant; *P < 0.05; **P < 0.01; ***P < 0.001.

qRT-PCR was used to determine the expression of seven core prognostic genes in Kasumi-1 and HL-60 cells treated with erastin or sulfasalazine for 24 h. After treatment with erastin or sulfasalazine, the mRNA expression levels of *ACSF2*, *SOCS1*, *SLC7A11*, and *AIFM2* increased, while those of *MYB* and *GPX4* decreased. Further, the gene expression level of *EIF2AK4* was almost unchanged. Of note, the decrease in *GPX4* in erastin- or sulfasalazine-treated Kasumi-1 and HL-60 cells and *MYB* in erastin-treated HL-60 cells was not statistically significant (**Figures 8A–D**). Western blot further verified the protein expression changes of SLC7A11 in Kasumi-1 and HL-60 cells after sulfasalazine treatment (**Figure 8E** and **Supplementary File 7**).

### Erastin and sulfasalazine block the SLC7A11/xCT-GSH-GPX4 pathway in Kasumi-1 and HL-60 cells

**Figure 9:**
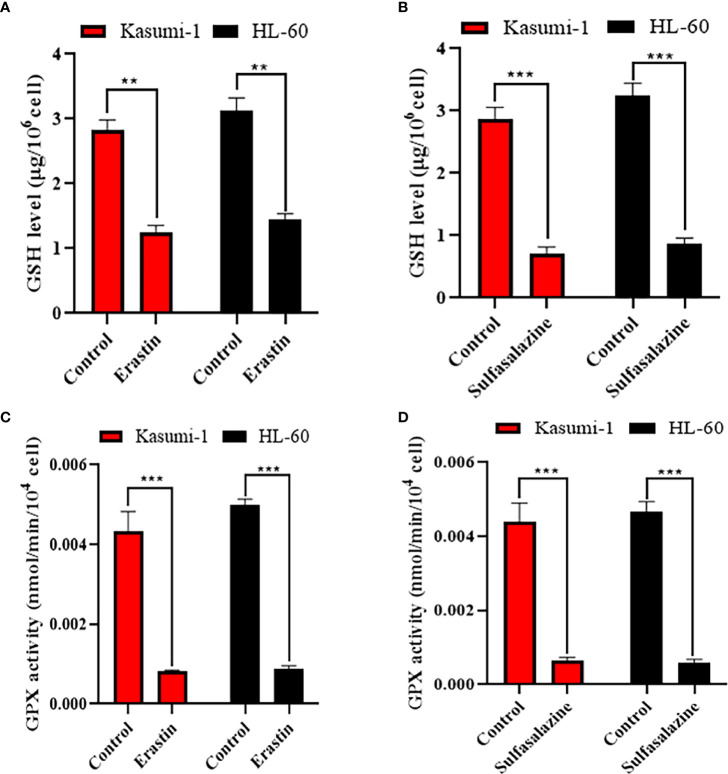
Erastin and sulfasalazine block the SLC7A11/xCT-GSH-GPX4 pathway in Kasumi-1 and HL-60 cells. **(A)** GSH levels were tested in Kasumi-1 and HL-60 cells cultured for 24 h under normal or 5µM erastin treatment conditions. **(B)** GSH levels were tested in Kasumi-1 and HL-60 cells cultured for 24 h under normal or 300µM sulfasalazine treatment conditions. **(C)** The activity of GPX4 was tested in Kasumi-1 and HL-60 cells cultured for 24 h under normal or 5µM erastin treatment conditions. **(D)** The activity of GPX4 was tested in Kasumi-1 and HL-60 cells cultured for 24 h under normal or 300µM sulfasalazine treatment conditions. NS, not significant; **P < 0.01; ***P < 0.001.

The SLC7A11/xCT-GSH/GPX4 pathway is a classical ferroptosis regulatory pathway. Therefore, we examined the GSH levels and GPX4 activity in Kasumi-1 and HL-60 cells. Both GSH levels (**Figures 9A, B**) and GPX4 activity (**Figures 9C, D**) were found to be significantly reduced after erastin- and sulfasalazine-induced ferroptosis of AML cells. Such findings suggest that the SLC7A11-GSH/GPX4 axis is critical for inducing the ferroptosis of Kasumi-1 and HL-60 cells by erastin and sulfasalazine.

## Discussion

Despite the rapid development of drugs for the treatment of leukemia in recent years, the problem of low OS has not considerably improved owing to the complex molecular mechanism and evolutionary advantageous cloning of AML (17). As a result, AML is still the most complex and one of the most challenging diseases. As a new form of cell death, ferroptosis has demonstrated potential in the treatment of various cancers, including AML (18, 19). In fact, researchers have linked ferroptosis-related genes with the prognosis of AML patients and constructed corresponding prognostic models to guide the treatment of AML patients (7, 8). However, these findings are biased as the researchers could not confirm whether certain key genes were involved in the regulation of AML ferroptosis in these models. Therefore, an effective prognostic prediction signature of ferroptosis-related genes was developed in this study and the changes in these genes after drug-induced ferroptosis of AML cells were explored. In addition, an attempt was made to identify potential therapeutic targets and mechanisms and closely link the prognosis of patients with AML with ferroptosis.

A total of 245 ferroptosis-related genes were obtained and patients with AML were randomly divided into training and validation groups at a ratio of 7:3. A signature containing seven ferroptosis-related genes with the lowest AIC values was also constructed using COX and LASSO regression analyses. All patients were divided into low- and high-risk groups according to their risk score. Based on the univariate and multivariate Cox regression analysis results for the training and validation groups, risk score is an independent risk factor affecting the prognosis of patients with AML. The t-SNE, PCA, and AUC results further confirmed the distinguishing ability and accuracy of the gene signature.

Further functional enrichment analysis revealed that the enriched pathways were involved in human T-cell leukemia virus 1 infection and many immune-related pathways. At present, evasion of the antitumor immune response is considered an important reason for the progression or relapse of AML, and immunotherapy has gradually become an important intervention for leukemia (20). Herein, the high-risk group was found to display higher expression levels of pDCs and Tregs. Studies have shown that pDCs promote tumor immune escape and are associated with poor prognosis (21). Treg-mediated immunosuppression is an important mechanism of tumor immune evasion and helps leukemia cells evade immune surveillance, ultimately promoting their progression (22). Owing to the complex interaction between ferroptosis and immunity (23), immune escape may be related to resistance to the ferroptosis of leukemia cells, thus leading to poor patient outcomes. However, further studies are needed to confirm these results.

We validated seven ferroptosis-related gene signatures *in vitro*. Based on our prognostic prediction model, *SLC7A11* was found to have the largest coefficient (coef) and the smallest *P* value, suggesting that it may be a key gene in our model. *SLC7A11* (xCT) and *SLC3A2* (alias 4F2) are part of the cystine/glutamate antiporter, system Xc^−^, whose activity is regulated by SLC7A11. SLC3A2 acts as a chaperone to maintain the stability of SLC7A11 (24). The antioxidant defense mediated by system Xc^−^ effectively protects cells and tissues from ferroptosis by transporting extracellular cystine into the cell. Once in the cell, cystine is rapidly converted to cysteine. Cysteine is the rate-limiting precursor for the synthesis of the important antioxidant, GSH (25). However, the observation of ferroptosis indicated that the inhibition of system Xc^−^ is the classic method of inducing ferroptosis. Among the known system Xc^−^ inhibitors, erastin is undoubtedly the most widely used (26). However, owing to the low metabolic stability and limited solubility of erastin, its application *in vivo* has been precluded (6). Therefore, sulfasalazine, a clinically approved drug that effectively inhibits system Xc^−^ and is used to treat inflammatory bowel disease and rheumatoid arthritis (5), was used.

Kasumi-1 and HL-60 cells were treated with different concentrations of erastin and sulfasalazine for 24 h. Based on the MTS results, erastin and sulfasalazine inhibited the proliferation of Kasumi-1 and HL-60 cells in a dose-dependent manner. Kasumi-1 and HL-60 cells were treated with four cell death inhibitors including ferroptosis inhibitor FER-1 and DFO, apoptosis inhibitor Z-VAD-FMK, necroptosis inhibitor necrostatin-1 and autophagy inhibitor chloroquine, in the form of elucidating the cell death involved. Interestingly, only the ferroptosis inhibitors markedly rescued erastin- and sulfasalazine-induced suppression of cell viability. In addition, the changes in the biochemical indicators of ferroptosis-related lipid peroxidation, including ROS and malondialdehyde (MDA) levels, were examined (4). In Kasumi-1 and HL-60 cells treated with erastin and sulfasalazine, the accumulation of oxidative products is a necessary condition for ferroptosis as high levels of ROS can cause oxidative damage and even spontaneous death of cancer cells. Based on these findings, erastin and sulfasalazine may induce the ferroptosis of AML cells.

qRT-PCR was performed to determine the expression of seven prognostic ferroptosis-related genes in Kasumi-1 and HL-60 cells. After treatment with erastin or sulfasalazine, the mRNA expression levels of the remaining six prognostic ferroptosis-related genes were changed, except for the mRNA expression levels of *EIF2AK4*. *SOCS1*, a suppressor of cytokine signaling 1, has been shown to interact with P53 to regulate ferroptosis (27), and its high expression has been associated with poor prognosis in patients with AML (28). According to previous studies, the ferroptosis inhibitor, DFO, can repair spinal cord injury by regulating the expression of ACSF2 (acyl-CoA synthetase family member 2) (29). EIF2AK4 (also known as GCN2), eukaryotic translation initiation factor 2 alpha kinase 4, and activated EIF2AK4 regulate ferroptosis by upregulating ATF4 under oxidative stress (30). MYB plays an important role in the regulation of cellular oxidative stress. Based on prior studies, MYB can promote erastin-induced ferroptosis in gastric cancer cells by interacting with CDO1 (31). AIFM2 was recently demonstrated to be an endogenous ferroptosis suppressor and is known as ferroptosis suppressor protein 1 (FSP1). Studies have shown that AIFM2 reduces ubiquinone to ubiquinol by regenerating NAD(P)H, which counteracts lethal lipid peroxidation through free-radical trapping (32). GPX4 (glutathione peroxidase 4) is an important peroxidase that is widely distributed in the body and is one of the main enzymes that catalyze the oxidation of GSH in the glutathione redox cycle. GSH can be used to reduce lipid hydroperoxides to lipid alcohols, thereby inhibiting ferroptosis (6).

The SLC7A11/xCT-GSH-GPX4 pathway, a classical regulatory pathway of ferroptosis, has been proven to be a powerful target for future treatment of various cancers, in which GSH plays an important role (33). In fact, its depletion will downregulate the activity of GPX4 and increase ROS levels (34). In the present study, high expression levels of SLC7A11 and GPX4 act as risk factors in patients with AML and are closely related to their poor prognosis. Therefore, AML cells were treated with the ferroptosis inducer, erastin, and the system Xc^−^ inhibitor, sulfasalazine. For the first time, sulfasalazine was found to induce ferroptosis. After erastin- and sulfasalazine-induced ferroptosis of AML cells, the mRNA and protein expression of *SLC7A11* increased, as revealed by the qRT-PCR and Western blot results; this finding is similar to that of previous studies on other tumors (35–37), suggesting that erastin and sulfasalazine may induce adaptive cellular responses in AML cells under stress conditions. Simultaneously, the mRNA expression level of *GPX4* decreased after erastin and sulfasalazine treatment; however, the difference was not statistically significant. As a phospholipid hydroperoxidase, the role of GPX4 in cells mainly depends on its enzymatic activity (38). It can reduce lipid peroxides to lipid alcohols through the active site, thereby maintaining intracellular homeostasis and preventing the accumulation of ROS, thus playing an important role in preventing the occurrence of ferroptosis (4, 39). As the rate-limiting precursor of GPX4 enzymatic activity, the change of GSH content plays a crucial role in GPX4 enzymatic activity (40). Therefore, following the methods used in previous studies, we examined changes in GSH levels and GPX4 activity in cells (6, 34). As expected, the GSH levels and GPX4 activity were significantly reduced. These results suggest that both erastin and sulfasalazine can induce the ferroptosis of AML cells by inhibiting system Xc^−^ activity, thereby reducing intracellular GSH levels and inhibiting GPX4 activity. Therefore, the SLC7A11/xCT-GSH-GPX4 pathway might be a key pathway that regulates the ferroptosis of AML cells. This study had certain limitations. First, only ferroptosis class I inducers (5) were used to confirm the expression changes of relevant ferroptosis genes; these inducers do not cover all possible ferroptotic changes in AML cells. Second, the reasons for the changes in the expression of genes besides *SLC7A11* and *GPX4* were not further explored; whether such genes can affect the prognosis of AML cells by regulating ferroptosis remains to be investigated. Third, whether the SLC7A11/xCT-GSH-GPX4 pathway plays an important role in the induction of ferroptosis in AML cells by sulfasalazine and erastin still needs further experiments to confirm. Fourth, whether erastin and sulfasalazine can induce other cell death types in AML cells still needs further experiments to investigate. Finally, further functional and *in vivo* experiments are still required to validate the accuracy of our model. Despite these limitations, to the best of our knowledge, this is the first study to combine the construction of a ferroptosis-related gene prognostic signature in AML with the induction of ferroptosis of AML cells. Equally important, we provided the first evidence that the Food and Drug Administration (FDA)-approved drug, sulfasalazine, can induce the ferroptosis of AML cells. Further, SLC7A11 may be a key regulator of the ferroptosis of AML cells.

## Conclusion

In conclusion, we identified and validated seven ferroptosis-related gene signatures (*SOCS1*, *ACSF2*, *MYB*, *EIF2AK4*, *AIFM2*, *SLC7A11*, and *GPX4*) with independent prognostic value. Based on further studies, *SLC7A11* and the SLC7A11/xCT-GSH-GPX4 pathway may be the respective key gene and underlying mechanism of erastin-and sulfasalazine-induced ferroptosis of AML cells. As sulfasalazine has been approved for use by the FDA, we will verify the ferroptosis-inducing mechanism of sulfasalazine in AML using *in vivo* experiments. Overall, our findings provide a new therapeutic strategy of targeting the ferroptosis of AML cells to improve patient outcomes.

## Data availability statement

The original contributions presented in the study are included in the article/**Supplementary Material**. Further inquiries can be directed to the corresponding author.

## Author contributions

ZZ and DL contributed to the conception and design. ZZ, XH, XH, XJ, HJ, YH, WW, YX and DL conducted the experiments. ZZ, XH, XH, XJ, HJ, YH, WW, YX and DHL collected and analyzed data. ZZ, DL, XH and XH edited and critically reviewed the manuscript. All authors contributed to the article and approved the final manuscript.

## Funding

This research was funded by the National Natural Science Foundation of China (approval number 82072355) and Postgraduate Innovation Project of Fujian Medical University (approval number 2020QH2015).

## Acknowledgments

We thank the FerrDb and TCGA databases for free and open access.

## Conflict of interest

The authors declare that the research was conducted in the absence of any commercial or financial relationships that could be construed as a potential conflict of interest.

## Publisher’s note

All claims expressed in this article are solely those of the authors and do not necessarily represent those of their affiliated organizations, or those of the publisher, the editors and the reviewers. Any product that may be evaluated in this article, or claim that may be made by its manufacturer, is not guaranteed or endorsed by the publisher.

## Supplementary material

Supplementary Figure 1 Correlation between the expression level of 7 ferroptosis-related genes and risk scores in the entire corhort. **(A–G)** SLC7A11, SOCS1, AIFM2, MYB, GPX4, ACSF2, and EIF2AK4, respectively. **P*< 0.05; ***P*< 0.01; ****P*< 0.001

Supplementary Figure 2
**(A, B)** *t*-Distributed stochastic neighbor embedding (t-SNE) analysis was performed in the training and validation groups, respectively. **(C, D)** Principal component analysis (PCA) was performed in training group and validation group, respectively.

Supplementary File 1 Random group by R language.

Supplementary File 4 MDA reference proposal.

Supplementary File 6 Evidence of polish.
