# Supplementary figures and images for: Comprehensive analysis of ferroptosis-related gene signatures as a potential therapeutic target for acute myeloid leukemia: A bioinformatics analysis and experimental verification
Source: Front Oncol. 2022 Aug 11;12:930654. doi: 10.3389/fonc.2022.930654 (PMC9406152; doi:10.3389/fonc.2022.930654)

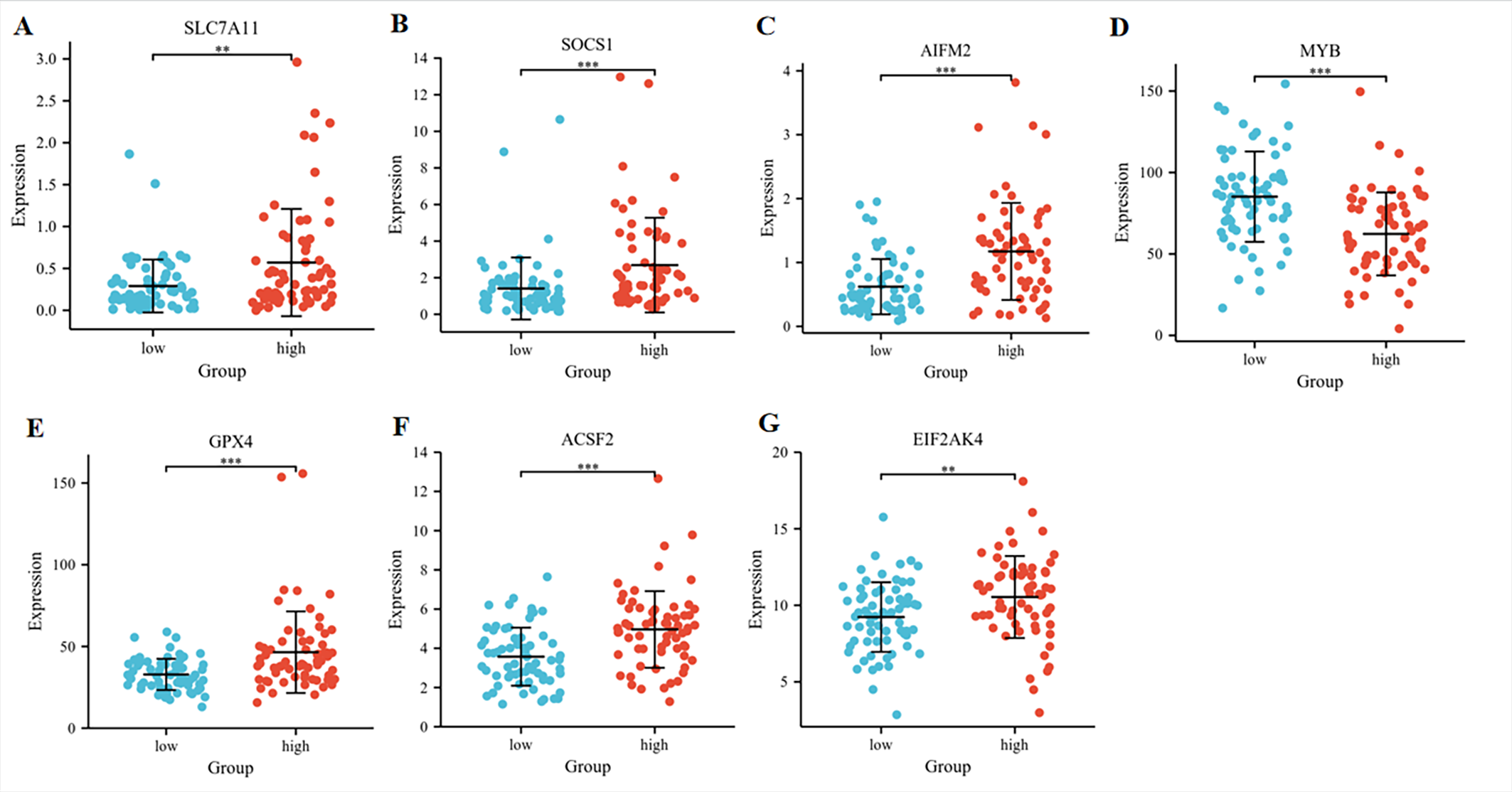

Supplement: Supplementary Figure 1 — Correlation between the expression level of 7 ferroptosis-related genes and risk scores in the entire corhort. (A–G) SLC7A11, SOCS1, AIFM2, MYB, GPX4, ACSF2, and EIF2AK4, respectively. *P< 0.05; **P< 0.01; ***P< 0.001 [file Image_1.tif]

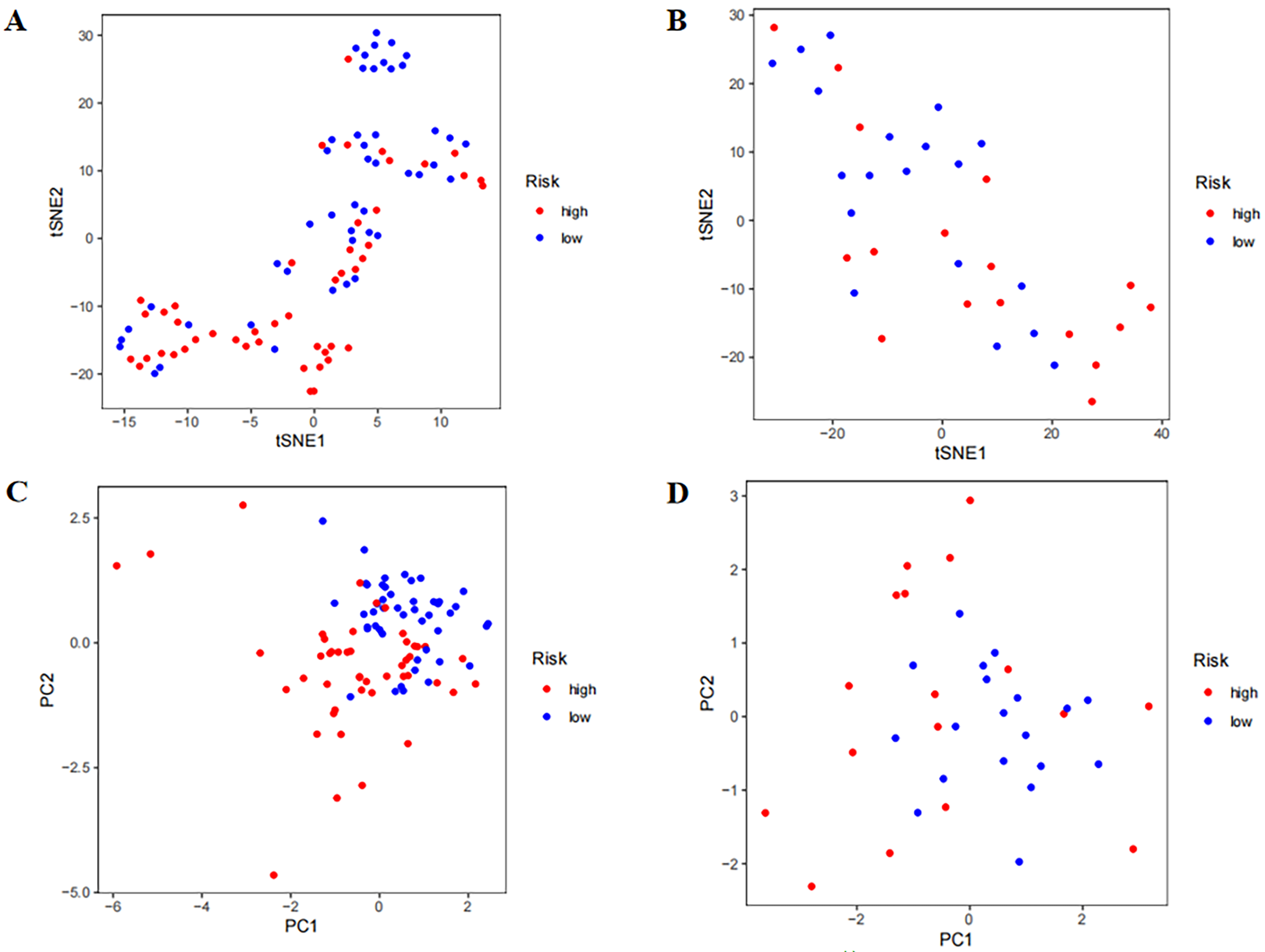

Supplement: Supplementary Figure 2 — (A, B) t-Distributed stochastic neighbor embedding (t-SNE) analysis was performed in the training and validation groups, respectively. (C, D) Principal component analysis (PCA) was performed in training group and validation group, respectively. [file Image_2.tif]
