# Supplementary material for: Comprehensive analysis of ferroptosis-related gene signatures as a potential therapeutic target for acute myeloid leukemia: A bioinformatics analysis and experimental verification
Source: Front Oncol. 2022 Aug 11;12:930654. doi: 10.3389/fonc.2022.930654 (PMC9406152; doi:10.3389/fonc.2022.930654)
Supplement: Supplementary File 1 — Random group by R language. [file Presentation_1.zip › Supplementary File 4 MDA reference proposal.PDF]

Beijing Solarbio Science & Technology Co., Ltd
Tel: 400-968-6088
Fax: 010-56371281
Http://www.solarbio.com

丙二醛（MDA）含量检测试剂盒说明书

微量法

注意：本产品试剂有所变动，请注意并严格按照该说明书操作。

货号：BC0025

规格：100T/96S

产品内容：使用前请认真核对试剂体积与瓶内体积是否一致，有疑问请及时联系索莱宝工作人员。

试剂名称	规格	保存条件
提取液	液体 110 mL×1 瓶	2-8℃保存
试剂一	液体 42 mL×1 瓶	2-8℃保存
试剂二	粉剂×2 瓶	2-8℃保存
试剂三	液体 12 mL×1 瓶	2-8℃保存

溶液的配制：

MDA 检测工作液的配制：临用前取 1 瓶试剂二加入 20mL 试剂一，溶解混匀，2-8℃保存一个月。MDA 检测工作液较难溶解，可以 70℃加热，并剧烈振荡以促进溶解，或者通过超声处理以促进溶解。

产品说明：

氧自由基作用于脂质的不饱和脂肪酸，生成过氧化脂质；后者逐渐分解为一系列复杂的化合物，其中包括丙二醛（MDA）。通过检测 MDA 的水平即可检测脂质氧化的水平。

丙二醛（MDA）在酸性和高温条件下，可以与硫代巴比妥酸（thiobarbituric acid，TBA）缩合，生成棕红色的三甲川（3,5,5-三甲基恶唑-2,4-二酮），其最大吸收波长在 532nm。进行比色后可估测样本中 MDA 的含量。

注意：实验之前建议选择 2-3 个预期差异大的样本做预实验。如果样本吸光值不在测量范围内建议稀释或者增加样本量进行检测。

需自备的仪器和用品：

可见分光光度计/酶标仪、水浴锅、台式离心机、可调式移液器、微量玻璃比色皿/96 孔板、研钵/匀浆器、冰和蒸馏水。

操作步骤：

一、样本处理（可适当调整待测样本量，具体比例可以参考文献）

1、 细菌或细胞样本的制备：收集细菌或细胞到离心管内，离心后弃上清；按照每 500 万细菌或细胞加入 1mL 提取液，超声波破碎细菌或细胞（功率 200W，超声 3s，间隔 10s，重复 30 次）；8000g 4℃离心 10min，取上清，置冰上待测。

2、 组织样本的制备：称取约 0.1g 组织，加入 1mL 提取液进行冰浴匀浆；8000g 4℃离心 10min，取上清，置冰上待测。

3、 低、中脂血血清（浆）等液体样本：直接检测。

4、 高脂血或者油脂类样本：取 40μL 样本和 80μL 无水乙醇混合（即样本被稀释 3 倍），涡旋震荡 5min 后使用。不同高脂肪类样本可能会有不同的稀释倍数，可以稀释不同倍数进行预实验以确定最佳稀释倍数。同时操作表中空白管的蒸馏水应以（33μL 蒸馏水+67μL 乙醇）代替。

二、测定步骤

1、 可见分光光度计/酶标仪预热 30min 以上，蒸馏水调零。

2、 按下表步骤加样：

试剂名称（μL）	测定管	空白管
MDA 检测工作液	300	300
蒸馏水	-	100
样本	100	-
试剂三	100	100

混合液在 100℃水浴中保温 60min 后（盖紧，防止水分散失），置于冰浴中冷却，10000g，常温，离心 10min。吸取 200μL 上清液于微量玻璃比色皿或 96 孔板中，测定各样本在 532nm 和 600nm 处的吸光度。分别计算 $\Delta A532=A532_{测定}-A532_{空白}$，$\Delta A600=A600_{测定}-A600_{空白}$，$\Delta A=\Delta A532-\Delta A600$（空白管只需做 1-2 次）。

注意高脂血或者油脂类样本在操作表中空白管的蒸馏水应以（33μL 蒸馏水+67μL 乙醇）代替

三、MDA 含量计算

A、按微量比色皿计算

（1）按照蛋白浓度计算

MDA 含量（nmol/mg prot）$=[\Delta A\times V_{反总}\div(\varepsilon\times d)\times 10^9]\div(Cpr\times V_{样本})\times F=32.258\times\Delta A\div Cpr$

（2）按照样本质量计算

MDA 含量（nmol/g 质量）$=[\Delta A\times V_{反总}\div(\varepsilon\times d)\times 10^9]\div(W\times V_{样本}\div V_{提取})\times F = 32.258\times\Delta A\div W$

（3）按照细菌或细胞数量计算

MDA 含量（nmol/10^4 cell）$=[\Delta A\times V_{反总}\div(\varepsilon\times d)\times 10^9]\div(500\times V_{样本}\div V_{提取})\times F =0.0645\times\Delta A$

（4）按照血清（浆）体积计算

MDA 含量（nmol/mL）$=[\Delta A\times V_{反总}\div(\varepsilon\times d)\times 10^9]\div V_{样本}\times F =32.258\times\Delta A$

V 反总：反应体系总体积，5×10^{-4}L；ε: MDA 摩尔吸光系数，1.55×10^5 L/mol/cm；V 样本：加入样本体积，0.1mL；d：比色皿光径，1cm；V 提取：加入提取液体积，1mL；Cpr：样本蛋白质浓度，mg/mL；W：样本质量，g；500：细胞或细菌总数，500 万；10^9：单位换算系数，1mol=10^9nmol；F：稀释倍数，高脂血或者油脂类样本需乘以稀释倍数。

B、按 96 孔板计算

（1）按照蛋白浓度计算

MDA 含量（nmol/mg prot）$=[\Delta A\times V_{反总}\div(\varepsilon\times d)\times 10^9]\div(Cpr\times V_{样本})\times F =53.763\times\Delta A\div Cpr$

（2）按照样本质量计算

MDA 含量（nmol/g 质量）$=[\Delta A\times V_{反总}\div(\varepsilon\times d)\times 10^9]\div(W\times V_{样本}\div V_{提取})\times F = 53.763\times\Delta A\div W$

（3）按照细菌或细胞数量计算

MDA 含量（nmol/10^4 cell）$=[\Delta A\times V_{反总}\div(\varepsilon\times d)\times 10^9]\div(500\times V_{样本}\div V_{提取})\times F =0.1075\times\Delta A$

（4）按照血清（浆）体积计算

MDA 含量（nmol/mL）$=[\Delta A\times V_{反总}\div(\varepsilon\times d)\times 10^9]\div V_{样本}\times F =53.763\times\Delta A$

V 反总：反应体系总体积，5×10^{-4}L；ε: MDA 摩尔吸光系数，1.55×10^5 L/mol/cm；V 样本：加入样本体积，0.1mL；d：96 孔板光径，0.6cm；V 提取：加入提取液体积，1mL；Cpr：样本蛋白质浓度，mg/mL；W：样本质量，g 500：细胞或细菌总数，500 万；10^9：单位换算系数，1mol=10^9nmol；F：稀释倍数，高脂血或者油脂类样

本需乘以稀释倍数。

注意事项：

1. 若发现检测样本吸光值过低，可以将沸水浴时间 60min 调整为 90min 或者更长，但同一实验中的 MDA 的检测都需要延长到同一时间以免引起误差。

实验实例：

1、取马血清按照测定步骤操作，用 96 孔板测得计算 $\Delta A532=A532_{测定}-A532_{空白}=0.078-0.047=0.031$，$\Delta A600=A600_{测定}-A600_{空白}=0.053-0.043=0.01$，$\Delta A=\Delta A532-\Delta A600=0.021$ 按体积计：

MDA 含量（nmol/mL）$=53.763\times\Delta A=1.129$ nmol/mL。

2、取 500 万 hela 细胞，加 1mL 提取液进行样本处理，离心取上清后按测定步骤操作，用 96 孔板测得计算 $\Delta A532=A532_{测定}-A532_{空白}=0.101-0.046=0.055$，$\Delta A600=A600_{测定}-A600_{空白}=0.043-0.043=0$，$\Delta A=\Delta A532-\Delta A600=0.055$，按照细菌或细胞数量计算：

MDA 含量（nmol/10^4 cell）$=0.1075\times\Delta A=0.006$nmol/$10^4$ cell

3、取 0.1g 小鼠肝脏，加 1mL 提取液进行样本处理，离心取上清后按测定步骤操作，用 96 孔板测得计算 $\Delta A532=A532_{测定}-A532_{空白}=0.196-0.047=0.149$，$\Delta A600=A600_{测定}-A600_{空白}=0.125-0.043=0.082$，$\Delta A=\Delta A532-\Delta A600=0.067$，按照样本质量计算：

MDA 含量（nmol/g 质量）$= 53.763\times\Delta A\div W=36.02$ nmol/g 质量

相关发表文献：

[1] QianYi Peng,YiMin Wang,CaiXia Chen,et al. Inhibiting the CD38/cADPR pathway protected rats against sepsis associated brain injury. Brain Research. January 2018;(IF2.929）

[2] Zhigang Chen, Qiaoling Yuan, Guangren Xu,et al. Effects of Quercetin on Proliferation and H2O2-Induced Apoptosis of Intestinal Porcine Enterocyte Cells. Molecules. 2018; (IF3.06)

[3] Huiwen Xiao, Yuan Li, Dan Luo,et al. Hydrogen-water ameliorates radiation-induced gastrointestinal toxicity via MyD88's effects on the gut microbiota. experimental and molecular medicine. January 2018;(IF4.743)

[4] Xuejuan Xia,Yuxiao,Xing,Guannan Li,e alc. Antioxidant activity of whole grain Qingke (Tibetan Hordeum vulgare L.) toward oxidative stress in d-galactose induced mouse model. Journal of Functional Foods. June 2018;(IF3.197)

[5] Qilong Wang, Guosheng Xiao, Guoliang Chen,et al. Toxic effect of microcystin-LR on blood vessel development. Toxicological & Environmental Chemistry. Feb 2019;(IF3.547)

[6] Zeyong Zhang,Huanhuan Liu,Ce Sun,et al. A C2H2 zinc-finger protein OsZFP213 interacts with OsMAPK3 to enhance salt tolerance in rice. Journal of Plant Physiology. October 2018;(IF2.825)

[7] Lijiao Gu, Hantao Wang, Hengling Wei,et al. Identification, Expression, and Functional Analysis of the Group IId WRKY Subfamily in Upland Cotton (Gossypium hirsutum L). Frontier in Immunology. November 2018;(IF4.259)

[8] Ping Shao,Pei Wang,Ben Niu,et al. Environmental stress stability of pectin-stabilized resveratrol liposomes with different degree of esterification. International Journal of Biological Macromolecules. November 2018;(IF4.784)

参考文献：

[1] Spitz D R , Oberley L W . An assay for superoxide dismutase activity in mammalian tissue homogenates[J]. Analytical Biochemistry,1989

[2] Masayasu M , Hiroshi Y . A simplified assay method of superoxide dismutase activity for clinical use[J]. Clinica Chimica Acta.

相关系列产品：

BC3590/BC3595　过氧化氢（H_2O_2）含量检测试剂盒

BC1090/BC1095　黄嘌呤氧化酶（XOD）活性检测试剂盒

BC0690/BC0695　葡萄糖氧化酶（GOD）活性检测试剂盒

BC1270/BC1275　蛋白质羰基含量检测试剂盒

BC1280/BC1285　二胺氧化酶（DAO）活性检测试剂盒

BC1290/BC1295　超氧阴离子含量检测试剂盒
