# Supplementary material for: Comprehensive analysis of ferroptosis-related gene signatures as a potential therapeutic target for acute myeloid leukemia: A bioinformatics analysis and experimental verification
Source: Front Oncol. 2022 Aug 11;12:930654. doi: 10.3389/fonc.2022.930654 (PMC9406152; doi:10.3389/fonc.2022.930654)
Supplement: Supplementary File 1 — Random group by R language. [file Presentation_1.zip › Supplementary File 5 The sequences of primers for real-time PCR assays..docx]

**Supplementary File 5. The sequences of primers for real-time PCR assays.**

| Gene | Sequence |
| --- | --- |
| GAPDH-F | GGTGTGAACCATGAGAAGTATGA |
| GAPDH-R | GAGTCCTTCCACGATACCAAAG |
| ACSF2-F | ATGAGAAGACACCAGAGCAGTT |
| ACSF2-R | GCACCGTACATCAGACACATC |
| SOCS1-F | GCACTTCCGCACATTCCGTT |
| SOCS1-R | CCATCTTCACGCTAAGGGCG |
| MYB-F | TCTCCAAGAACTCCTACACCATT |
| MYB-R | TCAGCAACAATTCCAGATTCATCA |
| EIF2AK4-F | GCCACAGAACTGCTCAAGAG |
| EIF2AK4-R | GTCGCTGTCATAGGTGTAATCG |
| AIFM2-F | CACTGACGTTCCTCCTGTCC |
| AIFM2-R | GTAGAGACGAACAGGTCCCG |
| SLC7A11-F | CCTGTTGTGTCCACCATCTC |
| SLC7A11-R | GATGAAGATTCCTGCTCCAATGA |
| GPX4-F | GAGGCAAGACCGAAGTAAACTAC |
| GPX4-R | CCGAACTGGTTACACGGGAA |
