# Supplementary material for: Comprehensive analysis of ferroptosis-related gene signatures as a potential therapeutic target for acute myeloid leukemia: A bioinformatics analysis and experimental verification
Source: Front Oncol. 2022 Aug 11;12:930654. doi: 10.3389/fonc.2022.930654 (PMC9406152; doi:10.3389/fonc.2022.930654)
Supplement: Supplementary File 1 — Random group by R language. [file Presentation_1.zip › Supplementary File 7 Changes in protein expression of SLC7A11 in Kasumi-1 and HL-60 cells after 300 μM sulfasalazine treatment conditions.docx]

Supplementary File 7 Changes in protein expression of SLC7A11 in Kasumi-1 and HL-60 cells after 300 µM sulfasalazine treatment conditions.

Figure 1-3, Changes in the protein expression level of SLC7A11 after 300 µM sulfasalazine (including the results of 3 replicates of MARK)


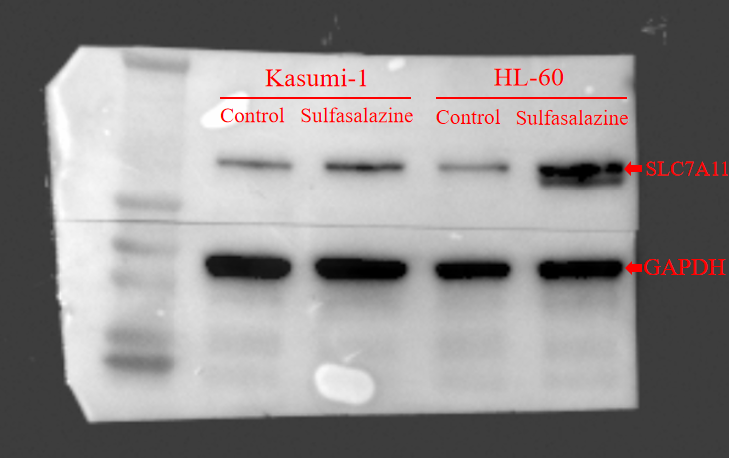


Figure 1 Changes in protein expression of SLC7A11 in Kasumi-1 and HL-60 cells after 300 µM sulfasalazine treatment


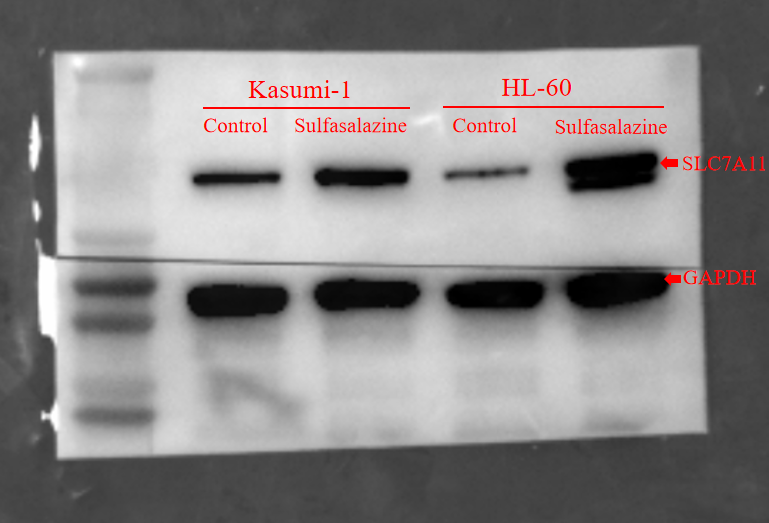


Figure 2 Changes in protein expression of SLC7A11 in Kasumi-1 and HL-60 cells after 300 µM sulfasalazine treatment


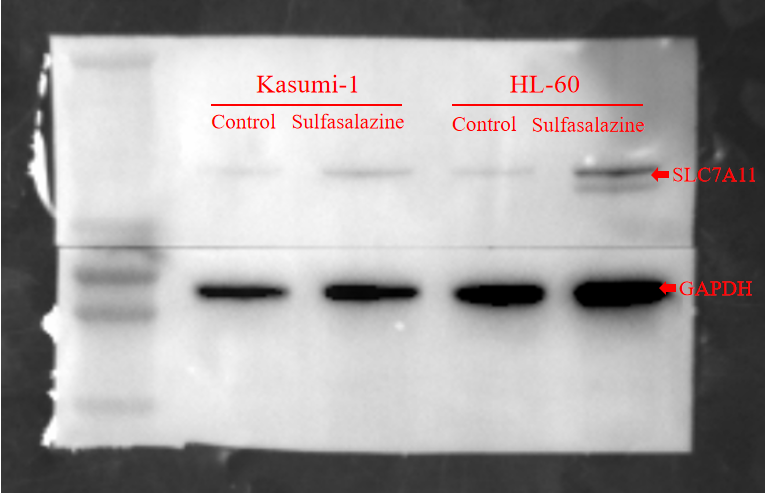


Figure 3 Changes in protein expression of SLC7A11 in Kasumi-1 and HL-60 cells after 300 µM sulfasalazine treatment
