# Supplementary material for: Comprehensive analysis of ferroptosis-related gene signatures as a potential therapeutic target for acute myeloid leukemia: A bioinformatics analysis and experimental verification
Source: Front Oncol. 2022 Aug 11;12:930654. doi: 10.3389/fonc.2022.930654 (PMC9406152; doi:10.3389/fonc.2022.930654)
Supplement: Supplementary File 1 — Random group by R language. [file Presentation_1.zip › Supplementary File 1 Random group by R language..docx]

**Supplementary File 1 Random group by R language.**

setwd("C:\\grouping")

rt=read.table("gene expression.txt",sep="\t",header=T,check.names=F)

library(caret)

inTrain<-createDataPartition(y=rt[,3],p=0.7,list=F)

train<-rt[inTrain,]

test<-rt[-inTrain,]

write.table(train,file="train group.txt",sep="\t",quote=F,row.names=F)

write.table(test,file="test grpup.txt",sep="\t",quote=F,row.names=F)
